# 111 Predictors of Social Participation Outcomes at Two Years after Burn Injury

**DOI:** 10.1093/jbcr/irad045.084

**Published:** 2023-05-15

**Authors:** Huan Deng, Lauren J Shepler, Diana Tenney, Mary D Slavin, Lewis Kazis, Colleen Ryan, Jeffrey Schneider, Barclay Stewart, Gretchen Carrougher, Karen Kowalske, Steven Wolf, Kaitlyn Linette Chacon

**Affiliations:** Spaulding Rehabilitation Hospital, Harvard Medical School, Boston, Massachusetts; Spaulding Rehabilitation Hospital, Harvard Medical School, Boston, Massachusetts; Burn Survivors of New England, Boston, Massachusetts; Department of Health Law, Policy and Management, Boston University School of Public Health, Boston, Massachusetts; Boston University School of Public Health, Rehabilitation Outcomes Center at Spaulding, Boston, Massachusetts; Shriners Children's-Boston, Massachusetts General Hospital, Harvard Medical School, Boston, Massachusetts; Spaulding Rehabilitation Hospital/Harvard Medical School/Rehabilitation Outcomes Center at Spaulding, Boston, Massachusetts; University of Washington, Seattle, Washington; University of Washington, Seattle, Washington; UT Southwestern Medical Center, Dallas, dallas, Texas; The University of Texas Medical Branch, Galveston, Texas; Spaulding Rehabilitation Hospital, Harvard Medical School, Charlestown, Massachusetts; Spaulding Rehabilitation Hospital, Harvard Medical School, Boston, Massachusetts; Spaulding Rehabilitation Hospital, Harvard Medical School, Boston, Massachusetts; Burn Survivors of New England, Boston, Massachusetts; Department of Health Law, Policy and Management, Boston University School of Public Health, Boston, Massachusetts; Boston University School of Public Health, Rehabilitation Outcomes Center at Spaulding, Boston, Massachusetts; Shriners Children's-Boston, Massachusetts General Hospital, Harvard Medical School, Boston, Massachusetts; Spaulding Rehabilitation Hospital/Harvard Medical School/Rehabilitation Outcomes Center at Spaulding, Boston, Massachusetts; University of Washington, Seattle, Washington; University of Washington, Seattle, Washington; UT Southwestern Medical Center, Dallas, dallas, Texas; The University of Texas Medical Branch, Galveston, Texas; Spaulding Rehabilitation Hospital, Harvard Medical School, Charlestown, Massachusetts; Spaulding Rehabilitation Hospital, Harvard Medical School, Boston, Massachusetts; Spaulding Rehabilitation Hospital, Harvard Medical School, Boston, Massachusetts; Burn Survivors of New England, Boston, Massachusetts; Department of Health Law, Policy and Management, Boston University School of Public Health, Boston, Massachusetts; Boston University School of Public Health, Rehabilitation Outcomes Center at Spaulding, Boston, Massachusetts; Shriners Children's-Boston, Massachusetts General Hospital, Harvard Medical School, Boston, Massachusetts; Spaulding Rehabilitation Hospital/Harvard Medical School/Rehabilitation Outcomes Center at Spaulding, Boston, Massachusetts; University of Washington, Seattle, Washington; University of Washington, Seattle, Washington; UT Southwestern Medical Center, Dallas, dallas, Texas; The University of Texas Medical Branch, Galveston, Texas; Spaulding Rehabilitation Hospital, Harvard Medical School, Charlestown, Massachusetts; Spaulding Rehabilitation Hospital, Harvard Medical School, Boston, Massachusetts; Spaulding Rehabilitation Hospital, Harvard Medical School, Boston, Massachusetts; Burn Survivors of New England, Boston, Massachusetts; Department of Health Law, Policy and Management, Boston University School of Public Health, Boston, Massachusetts; Boston University School of Public Health, Rehabilitation Outcomes Center at Spaulding, Boston, Massachusetts; Shriners Children's-Boston, Massachusetts General Hospital, Harvard Medical School, Boston, Massachusetts; Spaulding Rehabilitation Hospital/Harvard Medical School/Rehabilitation Outcomes Center at Spaulding, Boston, Massachusetts; University of Washington, Seattle, Washington; University of Washington, Seattle, Washington; UT Southwestern Medical Center, Dallas, dallas, Texas; The University of Texas Medical Branch, Galveston, Texas; Spaulding Rehabilitation Hospital, Harvard Medical School, Charlestown, Massachusetts; Spaulding Rehabilitation Hospital, Harvard Medical School, Boston, Massachusetts; Spaulding Rehabilitation Hospital, Harvard Medical School, Boston, Massachusetts; Burn Survivors of New England, Boston, Massachusetts; Department of Health Law, Policy and Management, Boston University School of Public Health, Boston, Massachusetts; Boston University School of Public Health, Rehabilitation Outcomes Center at Spaulding, Boston, Massachusetts; Shriners Children's-Boston, Massachusetts General Hospital, Harvard Medical School, Boston, Massachusetts; Spaulding Rehabilitation Hospital/Harvard Medical School/Rehabilitation Outcomes Center at Spaulding, Boston, Massachusetts; University of Washington, Seattle, Washington; University of Washington, Seattle, Washington; UT Southwestern Medical Center, Dallas, dallas, Texas; The University of Texas Medical Branch, Galveston, Texas; Spaulding Rehabilitation Hospital, Harvard Medical School, Charlestown, Massachusetts; Spaulding Rehabilitation Hospital, Harvard Medical School, Boston, Massachusetts; Spaulding Rehabilitation Hospital, Harvard Medical School, Boston, Massachusetts; Burn Survivors of New England, Boston, Massachusetts; Department of Health Law, Policy and Management, Boston University School of Public Health, Boston, Massachusetts; Boston University School of Public Health, Rehabilitation Outcomes Center at Spaulding, Boston, Massachusetts; Shriners Children's-Boston, Massachusetts General Hospital, Harvard Medical School, Boston, Massachusetts; Spaulding Rehabilitation Hospital/Harvard Medical School/Rehabilitation Outcomes Center at Spaulding, Boston, Massachusetts; University of Washington, Seattle, Washington; University of Washington, Seattle, Washington; UT Southwestern Medical Center, Dallas, dallas, Texas; The University of Texas Medical Branch, Galveston, Texas; Spaulding Rehabilitation Hospital, Harvard Medical School, Charlestown, Massachusetts; Spaulding Rehabilitation Hospital, Harvard Medical School, Boston, Massachusetts; Spaulding Rehabilitation Hospital, Harvard Medical School, Boston, Massachusetts; Burn Survivors of New England, Boston, Massachusetts; Department of Health Law, Policy and Management, Boston University School of Public Health, Boston, Massachusetts; Boston University School of Public Health, Rehabilitation Outcomes Center at Spaulding, Boston, Massachusetts; Shriners Children's-Boston, Massachusetts General Hospital, Harvard Medical School, Boston, Massachusetts; Spaulding Rehabilitation Hospital/Harvard Medical School/Rehabilitation Outcomes Center at Spaulding, Boston, Massachusetts; University of Washington, Seattle, Washington; University of Washington, Seattle, Washington; UT Southwestern Medical Center, Dallas, dallas, Texas; The University of Texas Medical Branch, Galveston, Texas; Spaulding Rehabilitation Hospital, Harvard Medical School, Charlestown, Massachusetts; Spaulding Rehabilitation Hospital, Harvard Medical School, Boston, Massachusetts; Spaulding Rehabilitation Hospital, Harvard Medical School, Boston, Massachusetts; Burn Survivors of New England, Boston, Massachusetts; Department of Health Law, Policy and Management, Boston University School of Public Health, Boston, Massachusetts; Boston University School of Public Health, Rehabilitation Outcomes Center at Spaulding, Boston, Massachusetts; Shriners Children's-Boston, Massachusetts General Hospital, Harvard Medical School, Boston, Massachusetts; Spaulding Rehabilitation Hospital/Harvard Medical School/Rehabilitation Outcomes Center at Spaulding, Boston, Massachusetts; University of Washington, Seattle, Washington; University of Washington, Seattle, Washington; UT Southwestern Medical Center, Dallas, dallas, Texas; The University of Texas Medical Branch, Galveston, Texas; Spaulding Rehabilitation Hospital, Harvard Medical School, Charlestown, Massachusetts; Spaulding Rehabilitation Hospital, Harvard Medical School, Boston, Massachusetts; Spaulding Rehabilitation Hospital, Harvard Medical School, Boston, Massachusetts; Burn Survivors of New England, Boston, Massachusetts; Department of Health Law, Policy and Management, Boston University School of Public Health, Boston, Massachusetts; Boston University School of Public Health, Rehabilitation Outcomes Center at Spaulding, Boston, Massachusetts; Shriners Children's-Boston, Massachusetts General Hospital, Harvard Medical School, Boston, Massachusetts; Spaulding Rehabilitation Hospital/Harvard Medical School/Rehabilitation Outcomes Center at Spaulding, Boston, Massachusetts; University of Washington, Seattle, Washington; University of Washington, Seattle, Washington; UT Southwestern Medical Center, Dallas, dallas, Texas; The University of Texas Medical Branch, Galveston, Texas; Spaulding Rehabilitation Hospital, Harvard Medical School, Charlestown, Massachusetts; Spaulding Rehabilitation Hospital, Harvard Medical School, Boston, Massachusetts; Spaulding Rehabilitation Hospital, Harvard Medical School, Boston, Massachusetts; Burn Survivors of New England, Boston, Massachusetts; Department of Health Law, Policy and Management, Boston University School of Public Health, Boston, Massachusetts; Boston University School of Public Health, Rehabilitation Outcomes Center at Spaulding, Boston, Massachusetts; Shriners Children's-Boston, Massachusetts General Hospital, Harvard Medical School, Boston, Massachusetts; Spaulding Rehabilitation Hospital/Harvard Medical School/Rehabilitation Outcomes Center at Spaulding, Boston, Massachusetts; University of Washington, Seattle, Washington; University of Washington, Seattle, Washington; UT Southwestern Medical Center, Dallas, dallas, Texas; The University of Texas Medical Branch, Galveston, Texas; Spaulding Rehabilitation Hospital, Harvard Medical School, Charlestown, Massachusetts; Spaulding Rehabilitation Hospital, Harvard Medical School, Boston, Massachusetts; Spaulding Rehabilitation Hospital, Harvard Medical School, Boston, Massachusetts; Burn Survivors of New England, Boston, Massachusetts; Department of Health Law, Policy and Management, Boston University School of Public Health, Boston, Massachusetts; Boston University School of Public Health, Rehabilitation Outcomes Center at Spaulding, Boston, Massachusetts; Shriners Children's-Boston, Massachusetts General Hospital, Harvard Medical School, Boston, Massachusetts; Spaulding Rehabilitation Hospital/Harvard Medical School/Rehabilitation Outcomes Center at Spaulding, Boston, Massachusetts; University of Washington, Seattle, Washington; University of Washington, Seattle, Washington; UT Southwestern Medical Center, Dallas, dallas, Texas; The University of Texas Medical Branch, Galveston, Texas; Spaulding Rehabilitation Hospital, Harvard Medical School, Charlestown, Massachusetts

## Abstract

**Introduction:**

People with burn injury often experience long-term social participation challenges. A previous study explored demographic and injury variables predicting social participation outcomes after burn injury. This study aims to further examine post-traumatic growth, and physical and psychological symptoms as predictors of social participation.

**Methods:**

This is a multi-center Burn Model System (BMS) study with a prospective cohort design. Adult BMS Database participants from July 2018 to April 2022 were included. Predictors were measured at 12- and 24-month after injury including Post-Traumatic Growth Inventory Short Form (PTGI-SF), Post-Traumatic Stress Disorder Checklist Civilian Version (PCL-C), Patient-Reported Outcomes Measurement Information System (PROMIS-29) Depression, Anxiety, Fatigue, Sleep Disturbance, Pain Interference and Heat Intolerance. Social participation outcomes were measured with Life Impact Burn Recovery Evaluation (LIBRE) Social Interactions and Social Activities at 24-month after injury. Multivariable regression analyses were conducted to assess the association between predictors and outcomes.

**Results:**

A total of 158 burn survivors were included with an average age of 47 years and a mean burn size of 19.5% total body surface area. Significant predictors included PCL-C and PROMIS-29 Depression for LIBRE Social Interactions, and Heat Intolerance, PROMIS-29 Pain Interference and Depression, and PCL-C for LIBRE Social Activities. See Table 1 for details of regression results.

**Conclusions:**

Overall, self-reported post-traumatic stress and depression are associated with limited social interactions while heat intolerance, pain interference, depression, and post-traumatic stress are related to fewer social activities.

**Applicability of Research to Practice:**

This study improves our understanding of the multiple and modifiable factors which influence social participation after burn injury.

